# Homology modelling, molecular docking, and molecular dynamics simulations reveal the inhibition of *Leishmania donovani* dihydrofolate reductase-thymidylate synthase enzyme by Withaferin-A

**DOI:** 10.1186/s13104-018-3354-1

**Published:** 2018-04-16

**Authors:** Bharadwaja Vadloori, A. K. Sharath, N. Prakash Prabhu, Radheshyam Maurya

**Affiliations:** 1 Department of Animal Biology, School of Life Sciences, University of Hyderabad, Prof. C.R. Rao Road, Gachibowli, Hyderabad, 500046 India; 2 Department of Biotechnology and Bioinformatics, University of Hyderabad, Hyderabad, India

**Keywords:** *Leishmania donovani*, DHFR-TS, *Withania somnifera*, Ashwagandha, Molecular docking, Withaferin-A, Methotrexate, Dihydrofolicacid, Antileishmanial drug

## Abstract

**Objective:**

Present in silico study was carried out to explore the mode of inhibition of *Leishmania donovani* dihydrofolate reductase-thymidylate synthase (*Ld* DHFR-TS) enzyme by Withaferin-A, a withanolide isolated from *Withania somnifera*. Withaferin-A (WA) is known for its profound multifaceted properties, but its antileishmanial activity is not well understood. The parasite’s DHFR-TS enzyme is diverse from its mammalian host and could be a potential drug target in parasites.

**Results:**

A 3D model of *Ld* DHFR-TS enzyme was built and verified using Ramachandran plot and SAVES tools. The protein was docked with WA-the ligand, methotrexate (MTX)-competitive inhibitor of DHFR, and dihydrofolic acid (DHFA)-substrate for DHFR-TS. Molecular docking studies reveal that WA competes for active sites of both *Hu* DHFR and TS enzymes whereas it binds to a site other than active site in *Ld* DHFR-TS. Moreover, Lys 173 residue of DHFR-TS forms a H-bond with WA and has higher binding affinity to *Ld* DHFR-TS than *Hu* DHFR and *Hu* TS. The MD simulations confirmed the H-bonding interactions were stable. The binding energies of WA with *Ld* DHFR-TS were calculated using MM-PBSA. Homology modelling, molecular docking and MD simulations of *Ld* DHFR-TS revealed that WA could be a potential anti-leishmanial drug.

**Electronic supplementary material:**

The online version of this article (10.1186/s13104-018-3354-1) contains supplementary material, which is available to authorized users.

## Introduction

Withaferin-A (WA) is among the most effective withanolide isolated from *W. somnifera* and has various effects like anti-bacterial, anti-inflammatory, anti-proliferative and potent anti-cancer properties [1–4]. Recently we demonstrated in vitro, that withanolides show potent anti-leishmanial activity [5] and a drastic reduction in parasite load in vivo [6].

**Fig. 1:**
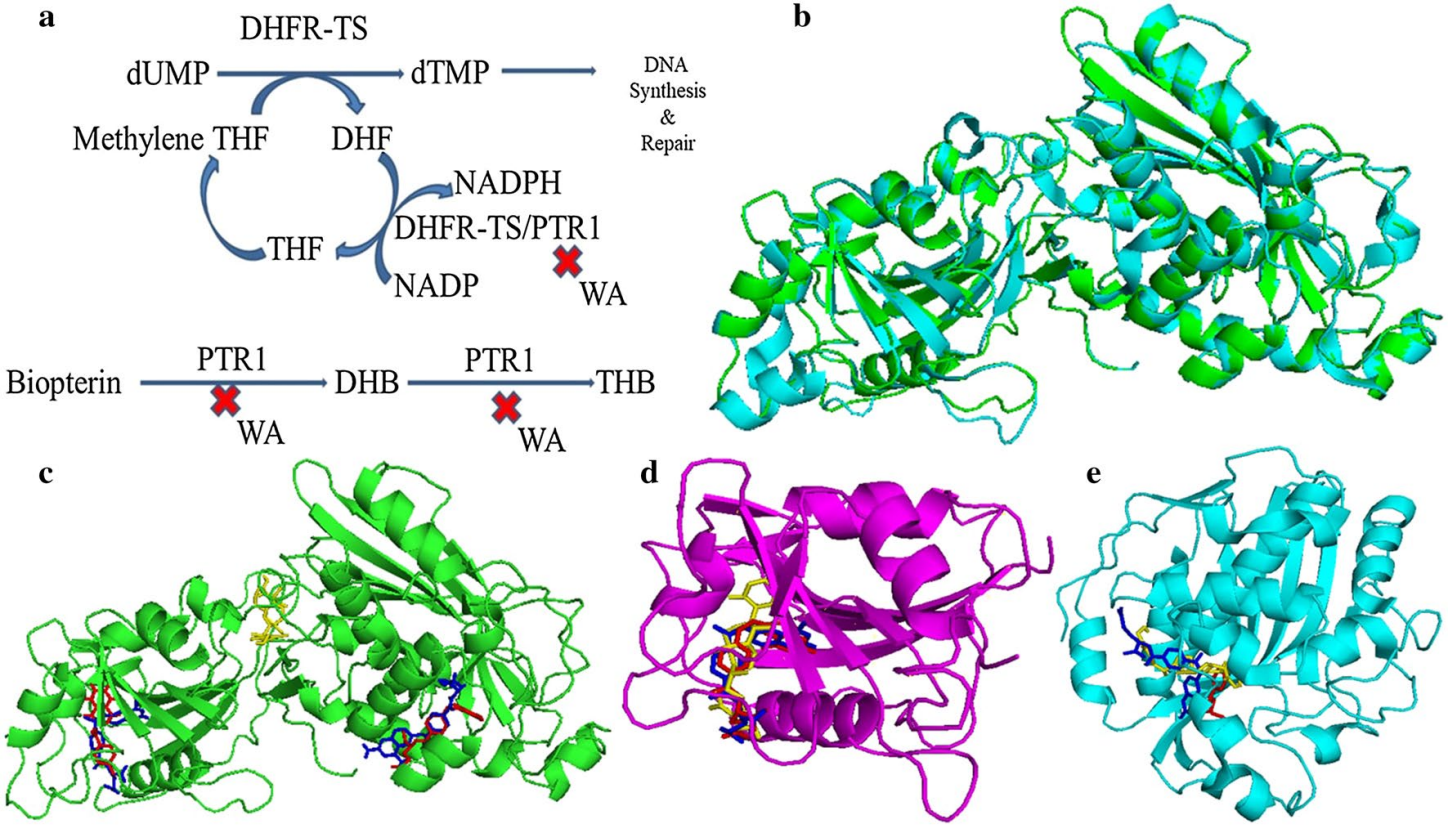
Folate biosynthesis pathway, homology modelling and molecular docking: **a** DHFR-TS synthesizes dTMP while converting methylene THF to DHF which is converted back to THF by DHFR-TS. PTR1 converts H2 biopterin to H4 biopterin. PTR1 can reduce both pterins and folates. WA inhibits both PTR1 and DHFR-TS enzymes. **b** Superimposed image of the template *T. cruzi* DHFR-TS chain A (PDB ID: 3INV) shown in blue and modeled *Ld* DHFR-TS shown in green. **c** Substrate DHFA (red) binds to two active sites of *Ld* DHFR-TS where an electrostatic channel is formed and substrate channeling between both the active sites is observed. Competitive inhibitor MTX (blue) competes with DHFA (red) and binds to two active sites of *Ld* DHFR-TS. Inhibitor WA (yellow) is binding to *Ld* DHFR-TS enzyme by blocking the electrostatic channel. **d** Substrate DHFA (red), Competitive inhibitor MTX (blue) and inhibitor WA (yellow) binding to *Hu* DHFR enzyme. **e** Substrate dUMP (red), inhibitors MTX (blue) and WA (yellow) binding to *Hu* TS enzyme

Availability of complete genome sequence of *Leishmania* opens new windows to identify a potential drug target [7]. Many enzymes of *Leishmania* are extensively explored as drug targets as they are diverse from mammalian hosts [8, 9]. Trypanosomatids including *Leishmania* are pteridine auxotrophs and require an exogenous source of folate/biopterin [10, 11]. Folate and biopterin are served as cofactors only in their fully reduced forms, H4-folate and H4-biopterin, respectively (Fig. 1a). In *Leishmania* DHFR along with TS forms DHFR-TS complex and occurs as a bifunctional enzyme [12–17]. However, as de novo biopterin synthetic pathway is absent, DHFR-TS shows no activity with biopterin [18–21]. Parasite obtains folates from the host and uses its DHFR-TS and PTR1 enzymes to reduce folates to active H4 forms [22–24].

Hence, folate biosynthesis enzymes can be potential drug targets and molecules which inhibit any enzyme of these pathways can be a safe antileishmanial drug. Our in silico study shows that WA inhibits multiple enzymes in folate biosynthesis pathway of *Leishmania* parasites.

## Main text

### Methods

#### Homology modeling

Amino acid sequences of *Ld* DHFR-TS, (accession no. CBZ31672.1, *Homo sapiens* or Human DHFR (*Hu* DHFR) (AAH71996.1) and *Homo sapiens* or Human TS (*Hu* TS) (NP_001062.1) were obtained from NCBI (http://www.ncbi.nlm.nih.gov). The similarity in sequences between host and parasite enzymes was identified using Clustal omega (https://www.ebi.ac.uk/Tools/msa/clustalo/). Template for structural modeling was identified using PDB-BLAST. Protein model was developed using SWISS-Model (https://swissmodel.expasy.org/) [25–28] and verified with Ramachandran plot, PROCHECK analysis, global model quality estimation (GMQE) score and qualitative model energy analysis (QMEAN) values [29].

#### Enzyme-ligand docking

The structures of WA, MTX and DHFA (PubChem CID 265237, 126941, 98792, respectively) were obtained from PubChem (https://pubchem.ncbi.nlm.nih.gov/) (Additional file 1: Fig. S1). Open Babel (http://openbabel.org/wiki/Main_Page) was used to obtain. pdbqt files. Molecular docking studies were carried out in Auto Dock Vina [30]. Initially, blind docking, was performed, followed by docking within restricted search space around the probable binding sites. Docking conformations were selected based on binding affinity. Pymol (https://www.pymol.org/) was used for visualization and graphical representations.

#### Drug-likeness prediction

Drug-likeness of WA [31, 32] was calculated using molsoft server (http://molsoft.com/mprop/). A drug-likeness plot and score were obtained. Swiss target was used to predict drug target class for Withaferin A. The server, using a combination of 2D and 3D similarity measures, compares the query molecule to a library of 280,000 compounds active on more than 2000 targets of five different organisms [33].

#### Molecular dynamic simulation

MD simulation of *Ld* DHFR-TS, *Hu* DHFR and *Hu* TS, and their WA complexes were performed in Gromacs 5.0. (http://www.gromacs.org/) [34]. The topological parameter of the ligand was obtained from ATB server (https://atb.uq.edu.au/) [35]. Initially, protein or its complex was kept in a cubic box filled with water using SPC/E water models. The system was energy minimized using GROMOS54a7 force field [36] and equilibrated at 300 K using V-rescale for 200 ps as NVT ensemble followed by equilibration at 1 atm pressure using Parrinello–Rahman algorithm as NPT ensemble for 200 ps. The equilibrated conformation was further extended for production simulation for 25 ns. LINCS algorithm was applied for bond constraints with distance cut-off using Verlet during simulation. Root mean square deviations of atomic coordinates during the simulation from their respective initial coordinates were calculated using the gmx_rms tool in Gromacs and binding energies were calculated using MM-PBSA [37].

### Results

#### Sequence alignment and homology modeling

The sequence similarity between *Hu* DHFR and *Ld* DHFR-TS was found to be 25.13%, and between *Hu* TS and *Ld* DHFR-TS, it was 54.63% suggesting that *Ld* DHFR-TS could be a valid drug target (Additional file 1: Figs. S2, S3). The amino acid sequence of *Ld* DHFR-TS was blasted against PDB-BLAST database for identifying an appropriate template for homology modeling. *T.cruzi* DHFR-TS showed 67.32% identity with the target protein and was selected as a template (Additional file 1: Fig. S4). Quality of the model generated by Swiss-model was verified using different tools (Fig. 1b) (Additional file 1: Table S1). The selected model showed 0.2% of residues in disallowed regions of Ramachandran plot (Additional file 1: Fig. S5, Table S2) with GMQE score of 0.82 and QMEAN score of − 2.25 (Additional file 1: Fig. S6).

The generated model is a homo-dimer protein of α + β class. The protein consists of 4β-sheets, 3βαβ units, 5β-hairpins, 19β-strands, 21α-helices (Additional file 1: Fig. S7). Similar numbers of secondary structural elements were found in *T. cruzi* DHFR-TS and RMSD between the template and generated model was calculated to be 0.625 Å.

#### Drug-likeness of Withaferin A

A compound to be considered as a drug should have ≤ 5 H-bond donors (HBD), ≤ 10 H-bond acceptors (HBA), molecular weight (MW) ≤ 500 Daltons, octanol–water partition coefficient (Log P) value between − 0.4 to + 5.6, and polar surface area (PSA) ≤ 140 Ǻ^2^ [38]. WA has 2HBDs, 6HBAs, MW of 470.27, Log P of 3.21, and PSA of 75.66 A^2^. The drug-likeness model score was 0.36 (Additional file 1: Table S3). Further, the frequency of drug target class as predicted by Swiss target prediction for WA is enzymes (40%) and kinases (33%).

#### Molecular docking studies

To know the active site of *Ld* DHFR-TS, it was first docked with its substrate DHFA and found that it has two active sites, one in DHFR and other in TS domain. TS active site is located 40 Å away from DHFR active site [38–40]. Asp 52, Arg 97 and Thr 180 of DHFR domain form H-bonds with DHFA and binding energy is − 29.3 kJ/mol. Arg 283, His 401, Gln 421, and Asn 433 of TS domain form H-bonds with DHFA and binding energy is − 31.8 kJ/mol.

MTX is a known competitive inhibitor of DHFR, hence *Ld* DHFR-TS was also docked with MTX. The results show that MTX binds at active sites (Fig. 1c). Ser 86 of DHFR domain forms H-bond with MTX and binding energy is − 33.1 kJ/mol. Arg 283, Glu 292, His 401, Gln 421 and Asn 433 of TS domain form H-bonds with MTX and binding energy is − 31.8 kJ/mol. The Binding site for MTX was compared with a 3D crystal structure of bifunctional *Tc* DHFR-TS in complex with MTX (3CL9) by superimposing on *Ld* DHFR-TS docked with MTX and RMSD of the ligand was found to be 0.625 Å. Likewise, crystal structure of mouse TS in ternary complex with *N*(4)-hydroxy-2′-deoxycytidine-5′-monophosphate and cofactor product, dihydrofolate (4EZ8), crystal structure of *Hu* TS, ternary complex with dUMP and tomudex (1i00) and *Hu* TS in complex with dUMP and MTX (5 × 66) were also used for superimposing and confirming the respective positions of ligands. RMSD values were 0.768, 0.806 and 0.669 Å respectively. Further, *Ld* DHFR-TS was docked with WA and Lys 173 forms an H-bond with WA. The binding energy of WA is − 42.7 kJ/mol and it binds in between both the active sites. It blocks the electrostatic channel of the enzyme (Fig. 1c).

Crystal structure of *Hu* DHFR (4m6k) was docked with WA and was superimposed with *Hu* DHFR ternary crystal complex of MTX and NADPH (1u72) and crystal structure of *Hu* DHFR complex of NADP+ and folate (4m6k). The results showed all three ligands viz. WA, DHFA, and MTX are binding in the same pocket. The ligand WA also competes for the active site and might be acting as a competitive inhibitor. The binding energy of WA is − 41.4 kJ/mol (Fig. 1d).

Crystal structure of *Hu* TS (1hzw) was docked with WA and later superimposed with *Hu* TS complex of dUMP and MTX (5x66). We observed that WA is binding at the same site like MTX. The residues Phe 80, His 196, Leu 221 and Asn 226 were forming H-bonds with WA and binding energy of WA was − 39.8 kJ/mol. The ligand WA was again competing for the active site and might be acting as a competitive inhibitor (Fig. 1e). Lys 173 forms an H-bond with WA. No H-bonding with WA was observed in *Hu* DHFR and Phe 80, His 196, Leu 221 and Asn 226 form H-bonds with WA in *Hu* TS. Although, WA is not binding in the active site of *Ld* DHFR-TS, it binds to human enzymes due to differences in the interacting residues.

The docking results of *Hu* DHFR and TS with WA suggest that WA competes for substrate binding sites of both human enzymes and act as competitive inhibitor. In case of *Ld* DHFR-TS, WA act as an uncompetitive inhibitor. The binding energy of *Ld* DHFR-TS with WA is higher than *Hu* DHFR and TS. Moreover, WA could be a better drug than MTX because of its high binding energy.

#### Molecular dynamic simulations of enzyme-inhibitor complexes

**Fig. 2:**
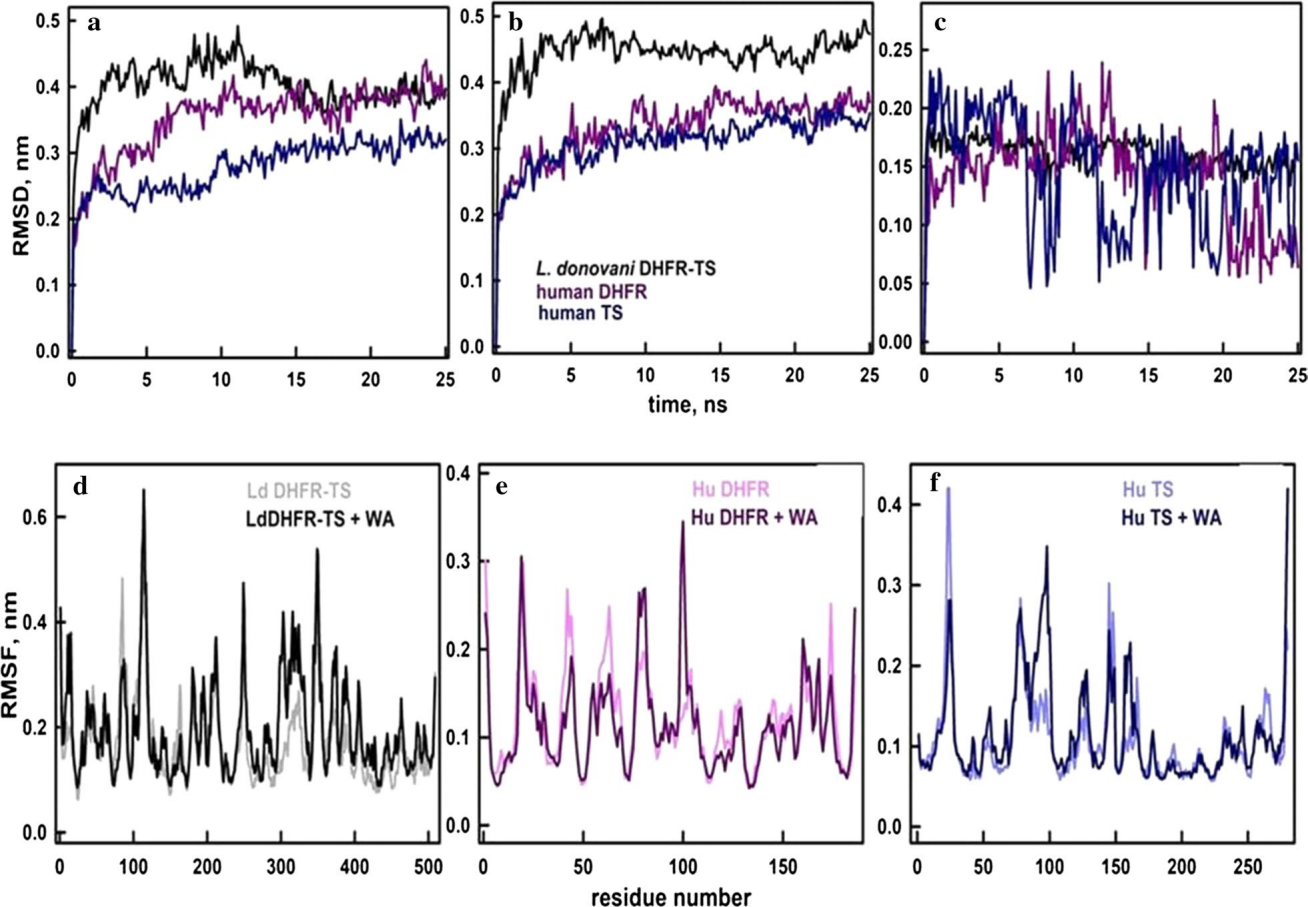
Molecular dynamics simulation: root mean square deviations (RMSD) of the proteins *Ld* DHFR-TS (black), *Hu* DHFR (red) and *Hu* TS (blue) **a** in the absence and **b** in the presence of WA. **c** Presents the RMSD of WA bound in different proteins. Root mean square fluctuations (RMSF) of Cα atoms of the residues of proteins: **d** *Ld* DHFR-TS, **e** *Hu* DHFR and **f** *Hu* TS in the absence and the presence of WA. The color codes are presented in the labels

To characterize the stabilizing interactions and to evaluate binding energies of WA with *Ld* DHFR-TS, *Hu* DHFR and *Hu* TS, MD simulation of proteins and protein-WA complexes were carried out. The analysis of root mean square deviations (RMSD) showed all proteins attained almost stable conformations (Fig. 2a–c) with comparable RMSD values. Addition of WA did not show much change in RMSD of *Hu* DHFR whereas RMSD of *Ld* DHFR-TS slightly increased. RMSD of ligand alone was around 0.15 nm in all proteins suggesting that bound conformation was stable. Further, root mean square fluctuations (RMSF) of individual residues were calculated by considering their Cα atoms as a reference (Fig. 2d–f). RMSF of β5-loop in DHFR domain and β1′ and β4′ loops in TS domain were found to increase slightly in the ligand-bound state of *Ld* DHFR-TS. The RMSF of β4 and β6 loops of DHFR domain reduced. In WA bound *Hu* DHFR, the fluctuations around β2, β3, and β6 loops reduced. In case of WA bound *Hu* TS protein, RMSF of β1 loop reduced whereas β3 increased. In all three proteins, changes in fluctuations were observed largely at sites away from ligand binding sites. Moreover, during binding of WA with *Ld* DHFR-TS, it was observed that WA formed H-bonding interactions with a backbone of F483 and side chains of Arg275, Asn199, and Asn231. Similarly, H-bonding interactions were identified between WA and backbone of Gly7 and side chain of Gln48 in *Hu* DHFR. WA formed H-bonding interactions with Arg163 and I1e 78 of *Hu* TS.

For further quantitative binding, energies of ligand were calculated by MM-PBSA using the last 10 ns of simulation data where RMSD of proteins were found to be more stable (Table 1). The analysis indicates that binding affinity of WA is more towards *Ld* DHFR-TS than *Hu* DHFR or *Hu* TS.

**Table 1:** Binding energy contributions of different interactions calculated using MM-PBSA

Types of energy (kJ/mol)	*Ld* DHFR-TS	*Hu* DHFR (kJ/mol)	*Hu* TS
Van der Waal energy	− 240.828 ± 14.111	− 152.257 ± 20.412	− 130.454 ± 11.349
Electrostatic energy	− 29.298 ± 9.846	− 24.299 ± 13.315	− 49.324 ± 14.361
Polar solvation energy	161.597 ± 20.010	95.665 ± 23.352	122.969 ± 30.393
Non-polar solvation energy	− 23.375 ± 1.019	− 16.934 ± 2.120	− 15.405 ± 2.536
Binding energy	− 131.904 ± 15.686	− 97.826 ± 24.200	− 72.214 ± 18.570

### Discussion

Interestingly*, Leishmania* dhfrts− mutants are unable to survive in mammalian host [41]. Deletion of PTR1 gene is lethal in promastigotes, indicating an essential role for unconjugated pteridines [20–23, 42]. PTR1 expression provides a potential ‘metabolic by-pass’ of DHFR-TS inhibition and allows a partial or complete reversal of anti-pteridine inhibition in the promastigote stage of parasites [20, 21]. PTR1 activity in *L. major* promastigotes is lower than in *L. donovani* and *L. mexicana. L. major* is more sensitive to MTX suggesting the role of PTR1 as a metabolic-bypass in *L. donovani* and *L. mexicana* [18, 19]. 3D structures of DHFR-TS and PTR1 of parasite and *Hu* DHFR have provided a strong base to design new inhibitors which are selective for parasite alone [43, 44].

Recently, we reported that WA inhibits *Ld* PTR1 enzyme activity and molecular docking studies of WA showed high binding affinity with PTR1. Enzyme assay with purified PTR-1 revealed that WA inhibits enzyme activity through uncompetitive mode [45]. The present molecular docking study reveals that the binding energy of WA with *Ld* DHFR-TS is higher than *Hu* DHFR, *Hu* TS enzymes and WA inhibits *Ld* DHFR-TS same as the PTR-1 enzyme. Thus it could be concluded that binding affinity of WA with multiple enzymes (DHFR-TS and PTR1) of folate biosynthesis pathway of parasites could make WA an effective anti-leishmanial drug.

### Limitation

Due to the lack of purified DHFR-TS enzyme, the current study could not include enzyme assay. However, enzyme assayed from parasite lysate with WA has shown the inhibition activity reported earlier [45].

## Additional file

**Additional file 1: Figure S1.** The structures of ligands: (A) Withaferin-A, (B) Methotrexate, (C) DHFA drawn using Chemdraw ultra version 12.0 software. **Figure S2.** Sequence identity between Hu DHFR and *Ld* DHFR-TS. Asterisks indicate identical amino acids. Dots and colons indicate conserved amino acid substitutions. Dashes indicate gaps. **Figure S3.** Sequence identity between Hu TS and *Ld* DHFR-TS. Asterisks indicate identical amino acids. Dots and colons indicate conserved amino acid substitutions. Dashes indicate gaps. **Figure S4.** Sequence identity between *Ld* DHFR-TS and *T.cruzi*chain A. Asterisks indicate identical amino acids. Dots and colons indicate conserved amino acid substitutions. Dashes indicate gaps. **Figure S5.** Ramachandran Plot: (A) Modelled *Ld* DHFR-TS and (B) reference *T. cruzi* DHFR-TS obtained using PROCHECK. **Figure S6.** Local quality estimate of (A) modelled *Ld* DHFR-TS and (B) reference *T.cruzi* DHFR-TS obtained from Swiss model. **Figure S7.** Secondary structures of (A) modelled *Ld* DHFR-TS and (B) reference T.cruzi DHFR-TS obtained from PDB sum. **Table S1.** Features of the generated *Ld* DHFR-TS model from Swiss model. **Table S2.** Ramachandran plot Statistics from PROCHECK results for modelled *Ld* DHFR-TS protein and reference *T. cruzi* DHFR-TS protein. **Table S3.** Drug likeness properties of WA from molsoft.

## Abbreviations

2D: two dimensional
3D: three dimensional
Å: Angstrom
ATB: automated topology builder
atm: atmosphere
DHFA: dihydrofolic acid
GMQE: global model quality estimation
HBA: hydrogen bond acceptors
HBD: hydrogen bond donors
Hu DHFR: human dihydrofolate reductase
Hu TS: human thymidylate synthase
K: Kelvin
*Ld* DHFR-TS: *Leishmania donovani* dihydrofolate reductase-thymidylate synthase
LINCS: LINear constraint solver
Log P: octanol–water partition coefficient
MDS: molecular dynamic simulations
MM-PBSA: molecular mechanics-Poisson–Boltzmann surface area
MTX: methotrexate
MW: molecular weight
nm: nano meters
ns: nano seconds
NCBI: National Center for Biotechnology Information
NPT ensemble: isothermal (constant temperature T)-isobaric (constant pressure P) ensemble
NVT ensemble: number of particles (N), absolute temperature (T) and volume (V) ensemble
PDB-BLAST: Protein Data Bank-Basic Local Alignment Search Tool
ps: pico seconds
PSA: polar surface area
PTR: pteridine reductase
QMEAN: qualitative model energy analysis
RMSD: root mean square deviation
RMSF: root mean square fluctuations
SAVES: structure analysis and verification server
SPC/E water models: extended simple point charge model
*Tc* DHFR-TS: *Trypanosoma cruzi* dihydrofolate reductase-thymidylate synthase
V-rescale: velocity rescale
WA: Withaferin A
