# Supplementary material for: Homology modelling, molecular docking, and molecular dynamics simulations reveal the inhibition of Leishmania donovani dihydrofolate reductase-thymidylate synthase enzyme by Withaferin-A
Source: BMC Res Notes. 2018 Apr 16;11:246. doi: 10.1186/s13104-018-3354-1 (PMC5902840; doi:10.1186/s13104-018-3354-1)

**Additional File 1:**

**Additional Table 1: Features of the generated *Ld* DHFR-TS model from Swiss model**

| Template | 3inv.1.A | Resolution | 2.37Å |
| --- | --- | --- | --- |
| Seq. Identity | 67.32 | Seq. Similarity | 0.50 |
| Oligostate | Homodimer | GMQE | 0.82 |
| Coverage | 0.99 | QMEAN4 | -2.25 |
| Found by | user  alignment | Description | Bifunctional  Dihydrofolate reductase  thymidylate synthase |
| Method | X ray |  |  |

**Additional Table 2: Ramachandran plot Statistics from PROCHECK results for modelled *Ld* DHFR-TS protein and reference *T. cruzi* DHFR-TS protein**

|  | No. of Residues | | Percentage | |
| --- | --- | --- | --- | --- |
|  | Modelled *Ld* DHFR-TS | Reference  *T. cruzi* DHFR-TS | Modelled *Ld*DHFR-TS | Reference *T. cruzi*  DHFR-TS |
| Most favoured regions [A, B, L] | 763 | 791 | 86.2% | 89.4% |
| Additional allowed regions [a, b, l, p] | 112 | 90 | 12.7% | 10.2% |
| Generously allowed regions [~a, ~b, ~l, ~p] | 8 | 4 | 0.9% | 0.5% |
| Disallowed regions [XX] | 2 | 0 | 0.2% | 0.0% |
| Non-glycine and non-proline residues | 885 | 885 | 100% | 100% |
| End-residues (excl. Gly, Pro) | 4 | 4 |  |  |
| Glycine residues | 64 | 76 |  |  |
| Proline residues | 64 | 62 |  |  |
| Total number of residues | 1017 | 1027 |  |  |

**Additional Table 3: Drug likeness properties of WA from molsoftTop of Form**

| Properties | WA |
| --- | --- |
| MF (Molecular formula) | C_28_H_38_O_6_ |
| MW(Molecular weight) | 470.27 |
| HBA (No. of Hydrogen bond acceptors) | 6 |
| HBD (No. of Hydrogen bond donors) | 2 |
| Mol Log P (Octanol/water partition coefficient) | 3.21 |
| Mol Log S (Solubility) | -4.07(in Log(moles/L)  or 39.60 (in mg/L) |
| Mol PSA (Molecular polar surface area) | 75.66 A^2^ |
| Mol Vol (Molecular volume) | 564.08 A^3^ |
| No. of Stereo centres | 11 |

**Additional Figure 1: The structures of ligands: A) Withaferin-A, B) Methotrexate, C) DHFA drawn using Chemdraw ultra version 12.0 software.**


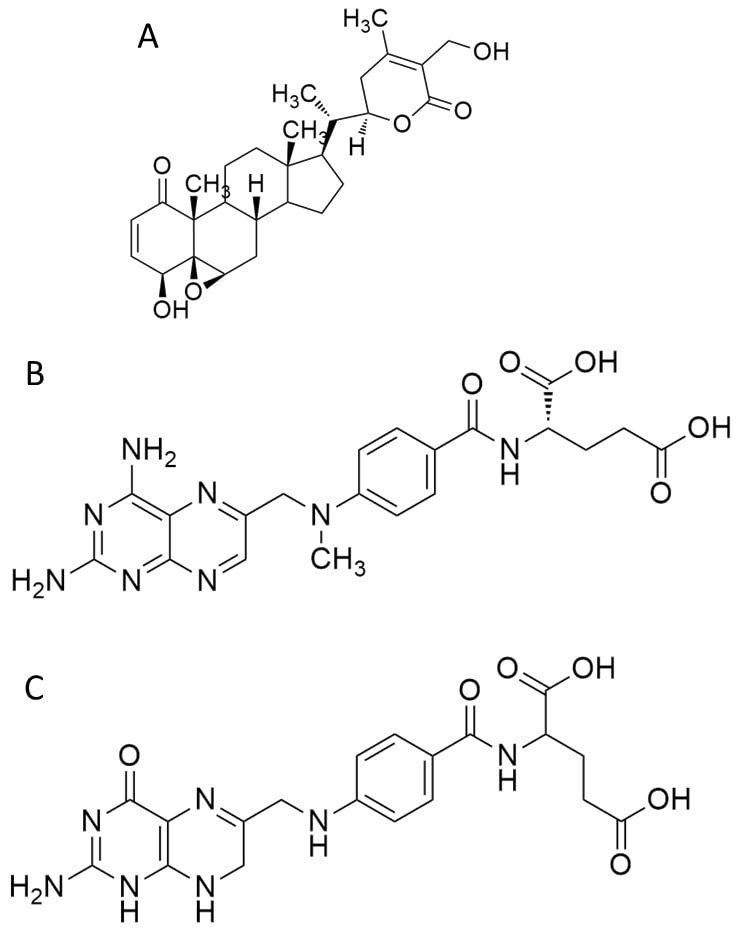


**Additional Figure 2:** Sequence identity between Hu DHFR and *Ld* DHFR-TS. Asterisks indicate identical amino acids. Dots and colons indicate conserved amino acid substitutions. Dashes indicate gaps.


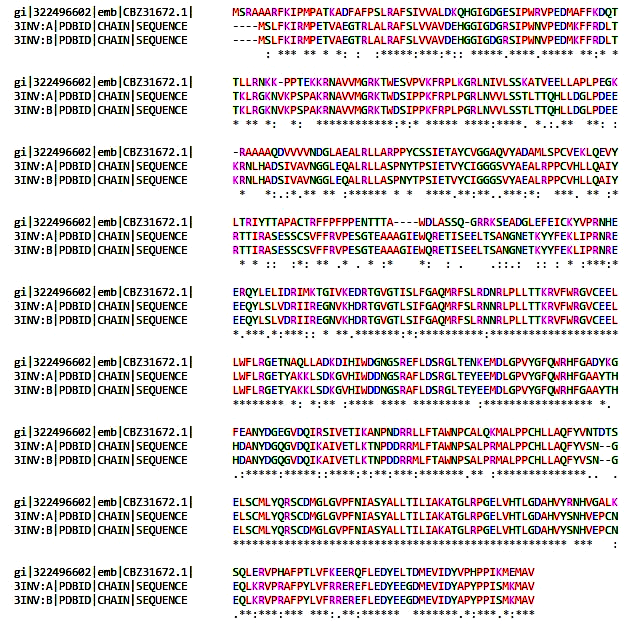


**Additional Figure 3**: Sequence identity between Hu TS and *Ld* DHFR-TS. Asterisks indicate identical amino acids. Dots and colons indicate conserved amino acid substitutions. Dashes indicate gaps.


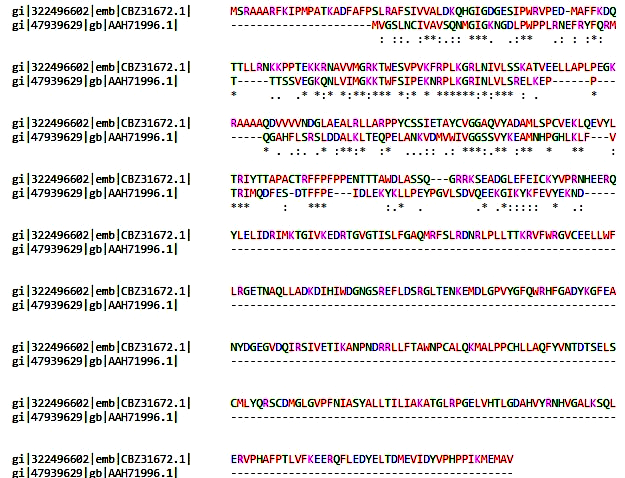


**Additional Figure 4**: Sequence identity between *Ld* DHFR-TS and *T.cruzi*chain A. Asterisks indicate identical amino acids. Dots and colons indicate conserved amino acid substitutions. Dashes indicate gaps.


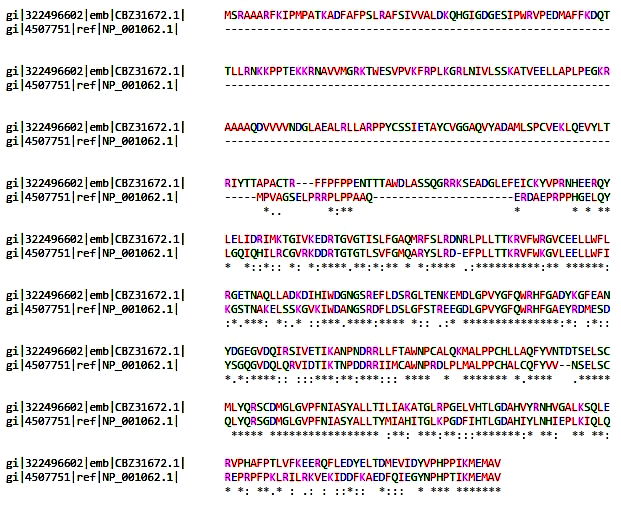


**Additional Figure 5:** Ramachandran Plot: A) Modelled *Ld* DHFR-TS and B) reference *T. cruzi* DHFR-TS obtained using PROCHECK.

**A**

**B**


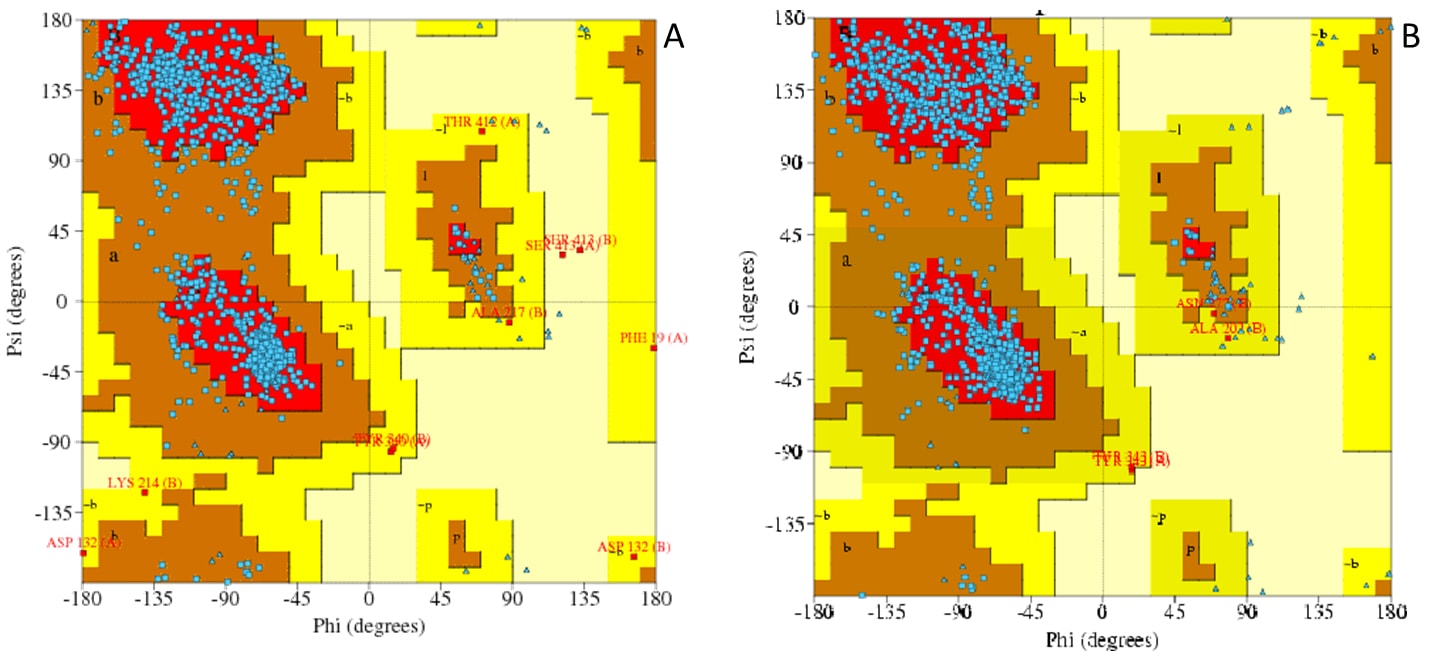


**Additional Figure 6**: Local quality estimate of A) modelled *Ld* DHFR-TS and B) reference *T.cruzi* DHFR-TS obtained from Swiss model.


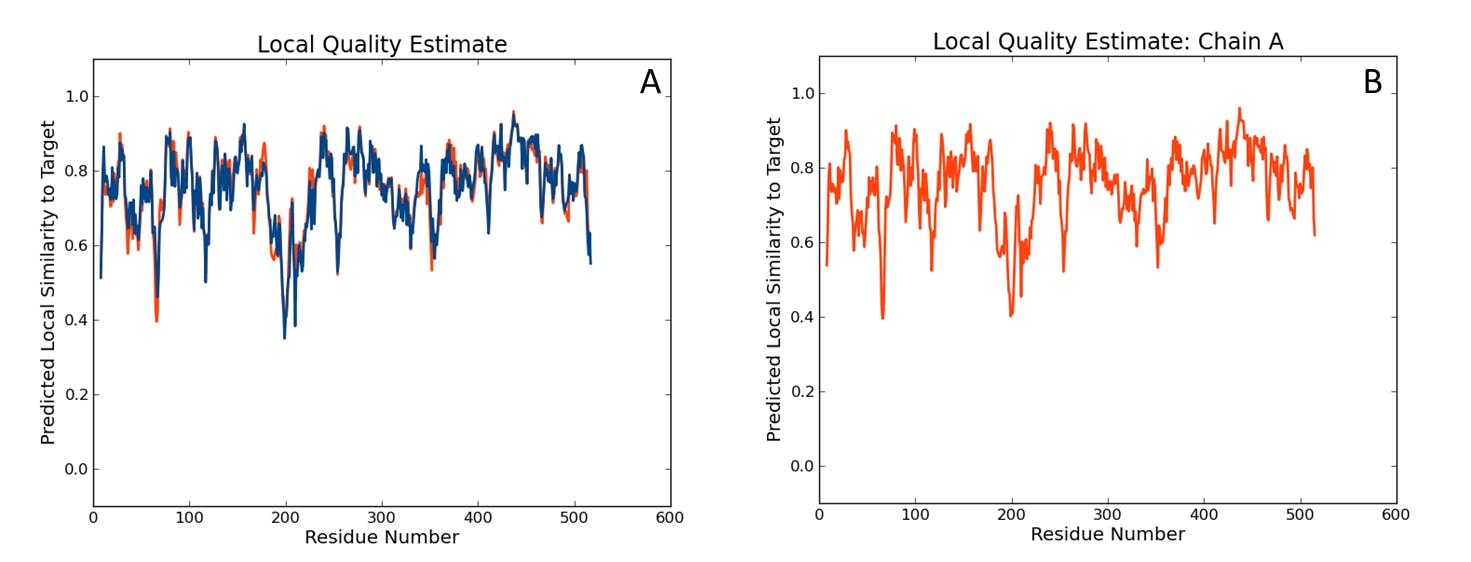


**Additional Figure 7**: Secondary structures of A) modelled *Ld* DHFR-TS and B) reference T.cruzi DHFR-TS obtained from PDB sum.


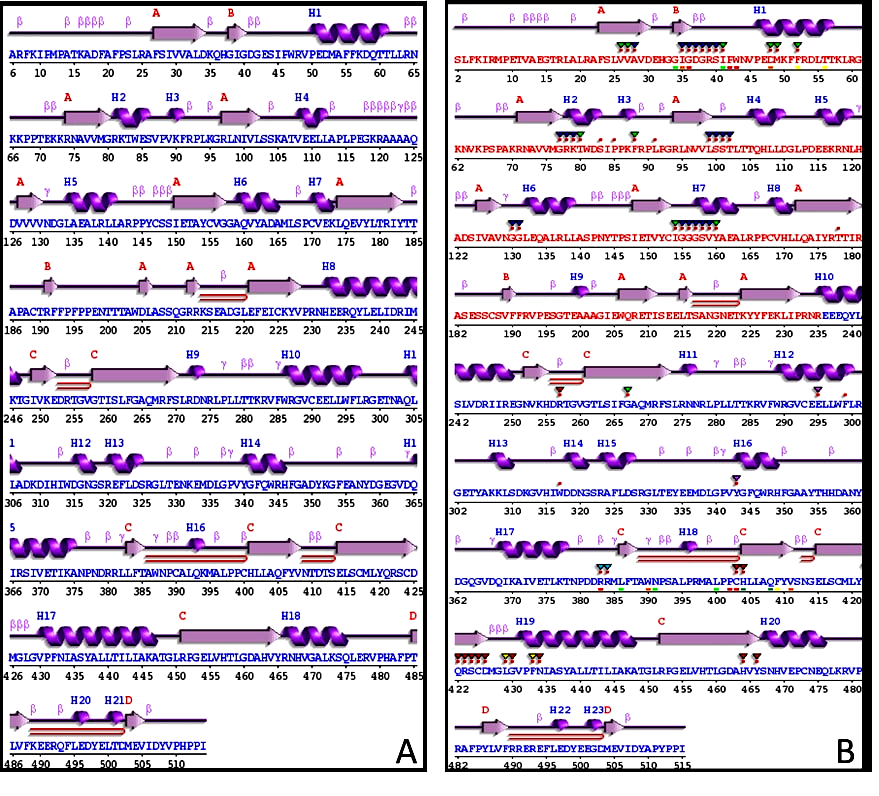

Supplement: Supplementary file 1 — Additional file 1: Figure S1. The structures of ligands: (A) Withaferin-A, (B) Methotrexate, (C) DHFA drawn using Chemdraw ultra version 12.0 software. Figure S2. Sequence identity between Hu DHFR and Ld DHFR-TS. Asterisks indicate identical amino acids. Dots and colons indicate conserved amino acid substitutions. Dashes indicate gaps. Figure S3. Sequence identity between Hu TS and Ld DHFR-TS. Asterisks indicate identical amino acids. Dots and colons indicate conserved amino acid substitutions. Dashes indicate gaps. Figure S4. Sequence identity between Ld DHFR-TS and T.cruzichain A. Asterisks indicate identical amino acids. Dots and colons indicate conserved amino acid substitutions. Dashes indicate gaps. Figure S5. Ramachandran Plot: (A) Modelled Ld DHFR-TS and (B) reference T. cruzi DHFR-TS obtained using PROCHECK. Figure S6. Local quality estimate of (A) modelled Ld DHFR-TS and (B) reference T.cruzi DHFR-TS obtained from Swiss model. Figure S7. Secondary structures of (A) modelled Ld DHFR-TS and (B) reference T.cruzi DHFR-TS obtained from PDB sum. Table S1. Features of the generated Ld DHFR-TS model from Swiss model. Table S2. Ramachandran plot Statistics from PROCHECK results for modelled Ld DHFR-TS protein and reference T. cruzi DHFR-TS protein. Table S3. Drug likeness properties of WA from molsoft. [file 13104_2018_3354_MOESM1_ESM.doc]
